# Efficacy, safety, and patient satisfaction of norditropin and sogroya in patients with growth hormone deficiency: a systematic review and meta-analysis of randomized controlled trials

**DOI:** 10.1007/s12020-024-03834-z

**Published:** 2024-04-24

**Authors:** Obieda Altobaishat, Mohamed Abouzid, Mostafa Hossam El Din Moawad, Abdulrahman Sharaf, Yazan Al-Ajlouni, Tungki Pratama Umar, Abdallah Bani-salameh, Mohammad Tanashat, Omar Abdullah Bataineh, Abdulqadir J. Nashwan

**Affiliations:** 1 Faculty of Medicine, Jordan University of Science and Technology, Irbid, Jordan; 2 Department of Physical Pharmacy and Pharmacokinetics, Faculty of Pharmacy, Poznan University of Medical Sciences, Rokietnicka 3 St., 60-806 Poznan, Poland; 3 Doctoral School, Poznan University of Medical Sciences, 60-812 Poznan, Poland; 4 Faculty of Pharmacy, Clinical Department Alexandria University, Alexandria, Egypt; 5 Faculty of Medicine, Suez Canal University, Isamailia, Egypt; 6 Department of Clinical Pharmacy, Salmaniya Medical Complex, Government Hospital, Manama, Bahrain; 7 School of Medicine, New York Medical College, New York, NY USA; 8 Faculty of Medicine, Universitas Sriwijaya, Palembang, Indonesia; 9 Division of Surgery and Interventional Science, University College London, London, United Kingdom; 10 Faculty of Medicine, Yarmouk University, Irbid, Jordan; 11 Hamad Medical Corporation, Doha, Qatar

**Keywords:** Sogroya, Norditropin, growth hormone deficiency, RCT, systematic review, meta-analysis

## Abstract

**Introduction:**

Growth hormone deficiency occurs when the pituitary gland does not produce enough growth hormone. Norditropin®, a recombinant human growth hormone, and Sogroya®, an albumin-binding growth hormone derivative, are prescribed for patients with growth hormone deficiency. This systematic review assesses the efficacy, safety, and patient satisfaction associated with Norditropin and Sogroya.

**Methods:**

We systematically searched PubMed, Web of Science, and Scopus databases to identify eligible comparative studies. All studies published until June 2023 were included in our analysis. Our outcomes for children included height velocity and height velocity standard deviation score. In contrast, adult outcomes included adverse events, insulin-like growth factor 1-standard deviation score (IGF-1 SDS), and the Treatment Satisfaction Questionnaire for Medication-9 (TSQM-9). Results are reported as odds ratio (OR) and mean difference (MD) with a 95% confidence interval (95% CI).

**Results:**

Ten studies involving 1058 participants (665 children and 393 adults) were included in the meta-analysis. In children, Norditropin at doses of 0.034 and 0.067 mg/kg/day was compared to Sogroya at doses of 0.04, 0.08, 0.16, and 0.24 mg/kg/week. The results showed that 0.034 mg/kg/day Norditropin had a favorable impact on height velocity (MD −2.01, 95% CI −3.7 to −2.12, *p* < 0.00001) and height velocity standard deviation score (Mean Difference −3.61, 95% CI −5.06 to −2.16, *p* < 0.00001) when compared to Sogroya 0.04 mg/kg/day. Other doses showed comparable results. In adults, the only significant side effect noted was rash, which favored Sogroya (OR 0.1, 95% CI 0.04–0.27, *p* < 0.00001). Additionally, IGF-1 SDS was significantly higher in the Sogroya group than in the Norditropin group (MD 0.25, 95% CI 0.02–0.48, *p* = 0.03). Furthermore, the overall score of the TSQM-9 questionnaire, which includes three domains: convenience, effectiveness, and satisfaction, was significantly higher in the Sogroya group compared to the Norditropin group (OR 6.36, 95% CI 3.92–8.8, *p* < 0.00001).

**Conclusion:**

Norditropin and Sogroya showed comparable efficacy and safety profiles, except for the prevalence of rash in the Norditropin group, and Sogroya has higher satisfaction among adults. More high-quality studies with more patients are required to confirm these results.

## Introduction

Growth Hormone Deficiency (GHD) is a medical disorder defined by insufficient production or release of growth hormone (GH) by the pituitary gland [1]. GHD can affect individuals of all ages, leading to physical symptoms such as retarded growth, delayed skeletal maturation, reduced bone density, short stature, decreased muscle mass, dyslipidemia, and elevated cardiovascular risks (including obesity and metabolic syndrome), as well as psychological issues like social discrimination, diminished self-worth, reduced functional capacity, and overall decreased quality of life [2–4]. The prevalence of GHD varies depending on the demographic studied. It is expected to occur in 1 in 4000 to 10,000 live births in children [5]. Research suggests that in the United States, males are twice as likely as females to undergo pediatric GH treatment [6]. A male predominance in referrals for short stature has been reported across age groups, potentially contributing to the observed disparity between males and females in the evaluation and treatment of short stature [7–9].

GHD is less common in adulthood, affecting about 1–1.5 in every 10,000 people [10]. Diagnosing GHD in childhood is challenging due to the absence of a gold standard and the generally poor performance of existing diagnostic tests [11]. The diagnosis is complicated by various factors affecting GHD, such as congenital anomalies of the pituitary gland or hypothalamus, brain tumors, infections, radiation treatment, or trauma to the hypothalamic-pituitary region [5]. Additionally, idiopathic GHD may develop in both children and adults. Treatment typically involves replacement therapy, which generally yields favorable outcomes [12].

Norditropin® (somatropin) is a daily GH produced by recombinant DNA technology administered to treat GHD [13]. It has been suggested to be beneficial in relieving GHD-related problems, which can help children reach their genetic height potential, especially if it is started early in the disease. A study recorded that most patients (78.5%) achieved a target near adult height NAH regardless of age at treatment initiation [14]. However, injection discomfort and the relative inconvenience of administering Norditropin (once-daily use) continue to be issues that limit therapy adherence in children and adults [15]. Furthermore, replacement therapy can cause cerebral hypertension, fluid retention, insulin resistance, scoliosis progression, and slipped capital femoral epiphysis [13].

As a contender to once-daily Norditropin, once-weekly Sogroya^®^ (somapacitan, a reversible albumin-binding GH derivative) is an emerging GH replacement therapy option. Somapacitan was well tolerated in a 26-week trial in patients with adult GHD, and no safety problems were detected (adverse effects were primarily mild and temporary) [16]. It has been claimed that once-weekly somapacitan is tolerated better than once-daily Norditropin. Meanwhile, after 26 and 52 weeks of treatment, once-weekly somapacitan (0.16 mg/kg/week) gave an equivalent efficacy with comparable safety and tolerability to daily GH in children with GHD [17]. Our systematic review aimed to assess the outcomes and patient satisfaction features of somapacitan and Norditropin, both of which are described for GHD patients.

## Methodology

### Protocol registration

This systematic review was conducted and reported per the Preferred Reporting Items for Systematic Reviews and Meta-Analyses (PRISMA) guidelines [18] and the Cochrane Handbook for systematic review and meta-analysis [19]. The review was registered with PROSPERO with the following ID (CRD42023444457).

### Data sources and search strategy

This study’s data sources and search strategy involved collaboration with a reference librarian and utilizing multiple databases. O.A. and M.T. thoroughly searched PubMed, Web of Science, and Scopus databases to ensure comprehensiveness. The search encompassed the entire timeframe from the databases’ inception to the present without imposing any temporal restrictions. In addition to database searches, a manual search was performed on the reference lists of retrieved journal articles, including systematic reviews. The search strategy employed a combination of Medical Subject Headings (MeSH terms) and relevant keywords related to the topic of interest. We used the following research query “((somapacitan) OR (Sogroya)) and ((Growth hormone) OR (GH) OR (Growth hormone therapy) OR (Norditropin) OR (somatropin))”

### Eligibility criteria

A PICO criterion was employed to include original research articles from peer-reviewed journals. The included studies consisted of randomized controlled trials (RCTs) conducted in English, specifically examining the efficacy and safety of somapacitan compared to daily GH in patients with GH deficiency. The eligible studies encompassed pediatric and adult participants diagnosed with GHD and treated with weekly somapacitan or daily growth hormone. The selected studies were required to measure and assess the impact of treatment on various parameters, including height, height SDS (standard deviation score), height velocity SDS, IGF-1 (insulin-like growth factor 1) SDS, TSQM-9 (Treatment Satisfaction Questionnaire for Medication), adverse events and injection site reactions.

Interventions not involving somapacitan were excluded from the study. This encompassed review articles, case reports, conference abstracts, and editorials. Studies with irrelevant data, particularly those lacking primary outcome data, were also excluded.

### Study selection

The review process was conducted using the Rayyan online software [20]. Two reviewers, O.A. and M.T., independently reviewed after removing duplicated records. Full-text screening was performed, and disagreements were resolved through discussions involving A.S.

### Data extraction

The data extraction process involved using a pre-designed extraction sheet to extract various data elements. The extracted data included baseline study characteristics such as the first author’s name, year of publication, country, journal name, and study design. Information regarding the included participants was also collected, including sample size, age, gender, weight, and BMI. Furthermore, outcome data were extracted, encompassing parameters such as height, height SDS (standard deviation score), height velocity SDS, IGF-1 SDS, TSQM-9, adverse events and injection site reactions. Two sets of two reviewers each (O.A.B, T.P.U, A.B-S, and Y.A-A.) conducted the data extraction process, and any discrepancies that emerged were resolved through discussion or consultation with the senior author.

### Risk of bias and quality assessment

Four reviewers (O.A.B, T.P.U, A.B-S, and Y.A-A) independently assessed the quality of the included studies in the research using the Cochrane RoB 2 tool [21]. The domains evaluated included the risk of bias resulting from the randomization process, the risk of bias due to deviation from the intended intervention, the risk of bias due to missing outcome data, the risk of bias in measuring the outcome, and the risk of bias in selecting the reported results. In cases of disagreement, the reviewers engaged in discussions and reached a consensus to resolve them.

### Statistical analysis

The statistical analysis was performed using RevMan v5.3 software [22]. The odds ratio (OR) was calculated to combine dichotomous results, while the mean difference (MD) was utilized to obtain continuous results. Both measures were accompanied by a 95% confidence interval (CI) and calculated using the random-effects model. The presence and extent of heterogeneity were assessed using the Chi-square and I-square tests, respectively. Consistent with the guidelines outlined in the Cochrane Handbook (chapter nine) [23], heterogeneity was deemed significant if the alpha level for the Chi-square test was below 0.1. Interpretation of the I-square test results was insignificant for 0–40 percent, moderate heterogeneity for 30–60 percent, and substantial heterogeneity for 50–90 percent.

## Results

**Fig. 1:**
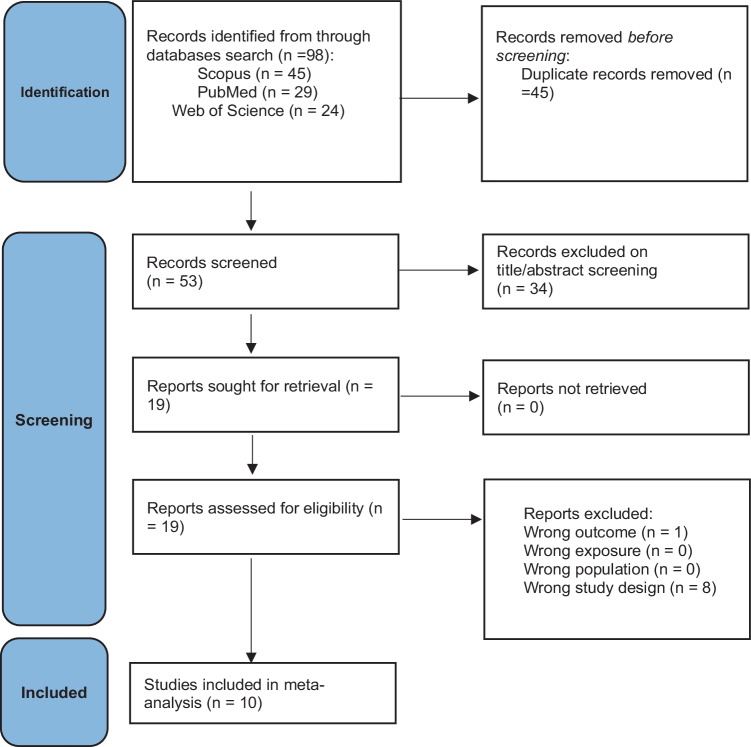
PRISMA flow chart of the screening process

Our search process in the included databases resulted in 98 articles, of which 53 were included in the title and abstract screening after removing duplicates. After that, full-text screening was done on a total of 19 articles, which resulted in 10 articles that were eligible for the final systematic review and meta-analysis (Fig. 1).

### Characteristics of the included studies

All included studies were of RCT design, whether phase two or three. The included studies compared the use of somapacitan in varying doses ranging from 0.02 to 0.24 mg/kg/week, while that of Norditropin was 0.034 or 0.067 mg/kg/day. The total number of participants was 1058 (665 children and 393 adults). We included studies comparing the safety and efficacy of somapacitan vs. Norditropin in adults and children, as shown in Table 1.

**Table 1:** Summary of the characteristics of the included studies and participants

Ref.	Study & year	Interventions	Sample size (N)	Age, Mean (SD)	Gender (M/F)	Weight, Mean (SD)	BMI, kg/m^2^, Mean (SD)	Organic, N (Total)	Aim of the study	Main findings
	Children									
[31]	Miller 2022	Somapacitan (0.16 mg/kg/wk)	132	6.4 (2.2)	(99/33)	16.7 (4.60)	15.7 (1.59)	17 (132)	To compare the efficacy and safety of somapacitan to that of daily Norditropin.	Over 52 weeks of treatment, somapacitan revealed equivalent efficacy to daily Norditropin, with comparable safety and mean IGF-1 SDS levels in treatment-naive children with GHD.
		Norditropin (0.034 mg/kg/d)	68	6.4 (2.4)	(50/18)	16.0 (4.95)	15.6 (1.38)	7 (68)		
[44]	Miller 2023	Somapacitan (0.16 mg/kg/wk)	132	6.4 (2.2)	(99/33)	16.7 (4.60)	15.7 (1.6)	17 (132)	Evaluate the efficacy and tolerability of somapacitan in children with GHD after 2 years of treatment and after switching from daily growth hormone.	Children with GHD who switched from daily Norditropin to somapacitan exhibited sustained efficacy and tolerance for 2 years. Patients and caregivers moving from daily Norditropin also stated that they preferred somapacitan.
		Norditropin (0.034 mg/kg/d)	68	6.4 (2.4)	(50/18)	16.0 (5.0)	15.6 (1.4)	7 (68)		
[17]	Sävendahl 2020	Somapacitan (0.04 mg/kg/wk)	14	5.8 (1.8)	(7/7)	14.2 (4.22)	15.3 (1.1)	0 (14)	Compare the efficacy, safety, and tolerability of once-weekly somapacitan against once-daily Norditropin.	After 26 and 52 weeks of treatment, once-weekly somapacitan 0.16 mg/kg/week gave the closest effectiveness match with equal safety and tolerability to daily Norditropin.
		Somapacitan (0.08 mg/kg/wk)	15	5.9 (1.8)	(10/5)	14.0 (3.54)	14.6 (1.1)	1 (15)		
		Somapacitan (0.16 mg/kg/wk)	14	6.1 (2.3)	(8/6)	14.9 (5.23)	15.1 (1.2)	1 (14)		
		Norditropin (0.034 mg/kg/d)	14	6.0 (2.0)	(9/5)	15.5 (5.03)	15.6 (1.4)	2 (14)		
[32]	Sävendahl 2022	Somapacitan (0.04 mg/kg/wk)	14	5.8 (1.8)	(7/7)	N/A	15.3 (1.1)	N/A	After three years of treatment, evaluate the efficacy, safety, and tolerability of once-weekly somapacitan.	Once-weekly somapacitan in children with GHD demonstrated maintained efficacy in all examined height-based outcomes over 3 years, with similar safety and tolerability to daily Norditropin.
		Somapacitan (0.08 mg/kg/wk)	15	5.8 (1.8)	(10/5)	N/A	14.6 (1.1)	N/A		
		Somapacitan (0.16 mg/kg/wk)	14	6.1 (2.3)	(8/6)	N/A	15.1 (1.2)	N/A		
		Norditropin (0.034 mg/kg/d)	14	5.9 (2.0)	(9/5)	N/A	15.6 (1.4)	N/A		
[45]	Sävendahl 2023	Pooled group	43	5.9 (2.0)	(25/18)	14.4 (4.3)	N/A	2 (43)	To evaluate somapacitan’s effectiveness and safety, as well as the disease/treatment burden, 4 years into treatment and 1 year after switching from daily Norditropin to somapacitan.	Patients who transitioned from daily Norditropin to somapacitan demonstrated equal efficacy and safety to those who continued somapacitan medication. Also, comparing once-daily injections to once-weekly injections may lessen the treatment load.
		Switched group	14	5.9 (2.0)	(9/5)	15.5 (5.0)	N/A	2 (14)		
[46]	Juul 2023	Somapacitan (0.16 mg/kg/wk)	12	6.13 (2.41)	(8/4)	15.05 (3.23)	14.66 (1.43)	N/A	To compare the efficacy, safety, and tolerability of three once-weekly somapacitan doses to daily Norditropin treatment in short children born SGA.	After 26 weeks of treatment, somapacitan 0.24 mg/kg/week appears to be the most effective, giving equal efficacy, safety, and tolerability as daily Norditropin 0.067 mg/kg/day in short children born SGA.
		Somapacitan (0.20 mg/kg/wk)	13	6.00 (2.46)	(9/4)	16.34 (5.07)	15.59 (1.23)	N/A		
		Somapacitan (0.24 mg/kg/wk)	12	6.10 (2.39)	(8/4)	14.87 (3.68)	14.86 (1.54)	N/A		
		Norditropin (0.035 mg/kg/d)	12	6.29 (2.84)	(6/6)	15.19 (4.14)	14.84 (1.31)	N/A		
		Norditropin (0.067 mg/kg/d)	13	5.92 (2.44)	(9/4)	14.78 (3.66)	14.81 (1.27)	N/A		
[34]	Battelino 2017	Somapacitan (0.02 mg/kg/wk)	6	8.25 (1.45)	(4/2)	24.62 (7.21)	15.85 (2.02)	0 (6)	To compare the safety, local tolerability, pharmacodynamics, and pharmacokinetics of once-weekly somapacitan, a reversible, albumin-binding GH derivative, to once-daily Norditropin in children with GHD.	When provided to pre-pubertal children with GHD, single doses of somapacitan in the dose range of 0.02–0.16 mg/kg were well tolerated. Also, no clinically significant safety signals were linked to somapacitan, nor were any immunogenicity issues detected.
		Somapacitan (0.04 mg/kg/wk)	6	8 (1.47)	(1/5)	27.72 (4.62)	17.4 (2.49)	4 (6)		
		Somapacitan (0.08 mg/kg/wk)	6	8.5 (1.19)	(5/1)	26.65 (4.42)	16.65 (1.33)	1 (6)		
		Somapacitan (0.16 mg/kg/wk)	6	8.75 (1.45)	(5/1)	27.47 (5.77)	15.47 (1.36)	2 (6)		
		Norditropin (0.03 mg/kg/d)	8	8.5 (1.44)	(8/0)	27.75 (6.59)	16.2 (1.30)	2 (8)		
	Adults									
[47]	Otsuka 2020	Somapacitan: Starting doses were as follows: 1.5 mg/week for adults 18–60 years of age, 2.0 mg/week for females on oral estrogen, and 1.0 mg/week for patients aged >60 years	46	54.1 (12.1)	(24/22)	69.4 (22.7)	26.4 (6.7)	4 (46)	To compare the safety and efficacy of once-weekly somapacitan versus daily GH in Japanese patients with adult GHD over 52 weeks.	Treatment was well tolerated in both groups, with no unexpected safety findings. Also, Somapacitan and daily GH affected adipose tissue, which was comparable in people with adult GHD.
		Norditropin: Starting doses were as follows: 0.2 mg/week for adults 18–60 years of age, 0.3 mg/week for females on oral estrogen, and 0.1 mg/week for patients aged >60 years	16	49.3 (11.5)	(9/7)	67.9 (12.0)	24.8 (3.7)	1 (16)		
[16]	Johannsson 2018	Somapacitan: After the dose titration period, the mean somapacitan dose (SD) was 1.96 (1.45) mg/week in the 18 weeks of fixed-dose treatment, compared with a starting dose of 1.5 mg/week.	61	48.1 (16.2)	(33/28)	82.1 (17.6)	28.6 (5.0)	18 (61)	To compare the safety of once-daily Norditropin and once-weekly somapacitan. Also, there was an evaluation of local tolerability and treatment satisfaction.	Somapacitan was well tolerated in this 26-week trial in patients with adult GHD; no safety concerns were found. Also, As opposed to daily Norditropin, once-weekly somapacitan was more practical.
		Norditropin: After the dose titration period, the mean dose after titration was 0.20 (0.14) mg/day, which was similar to the starting dose	31	51.7 (17.1)	(17/14)	81.0 (21.8)	28.5 (5.6)	7 (31)		
[48]	Johannsson 2020	Somapacitan: Starting doses were as follows: patients aged 23 to 60 years, 1.5 (0.214); patients aged > 60 years: 1.0 (0.143); and female patients on oral estrogen irrespective of age: 2.0 (0.286)	120	44.6 (14.3)	(58/62)	76.2 (21.0)	27.9 (6.3)	17 (120)	To compare somapacitan, a once-weekly reversible albumin-binding GH derivative, to placebo regarding efficacy and safety.	The overall therapy effects and safety of somapacitan were consistent with the known effects and safety of Norditropin replacement for up to 86 weeks of treatment, and it demonstrated superiority over placebo in adult GHD patients. Also, in adult GHD, somapacitan might be a good substitute for daily Norditropin.
		Norditropin: Starting doses were as follows: patients aged 23 to 60 years, 0.2; patients aged > 60 years: 0.1; and female patients on oral estrogen irrespective of age: 0.3	119	45.7 (15.3)	(58/61)	76.0 (22.7)	27.7 (6.2)	12 (119)		

*GH* growth hormone, *GHD* growth hormone deficiency, *IGF-1* insulin-like growth factor 1, *SDS* standard deviation score, *SGA* small for gestational age

### Risk of bias assessment

**Fig. 2:**
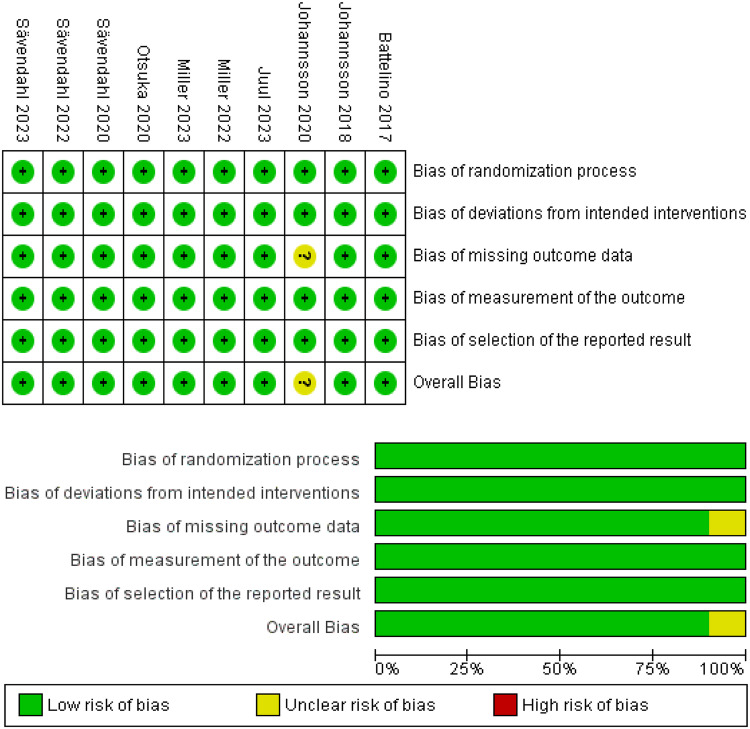
Quality assessment of risk of bias in the included trials. The upper panel presents a schematic representation of risks (low = red, unclear = yellow, and high = red) for specific types of biases of each of the studies in the review. The lower panel presents risks (low = red, unclear = yellow, and high = red) for the subtypes of biases of the combination of studies included in this review

According to RoB 2, none of the included studies had a high risk of bias, 9 had a low risk, and only one had some concerns (Fig. 2).

## Outcomes

### Children population

**Fig. 3:**
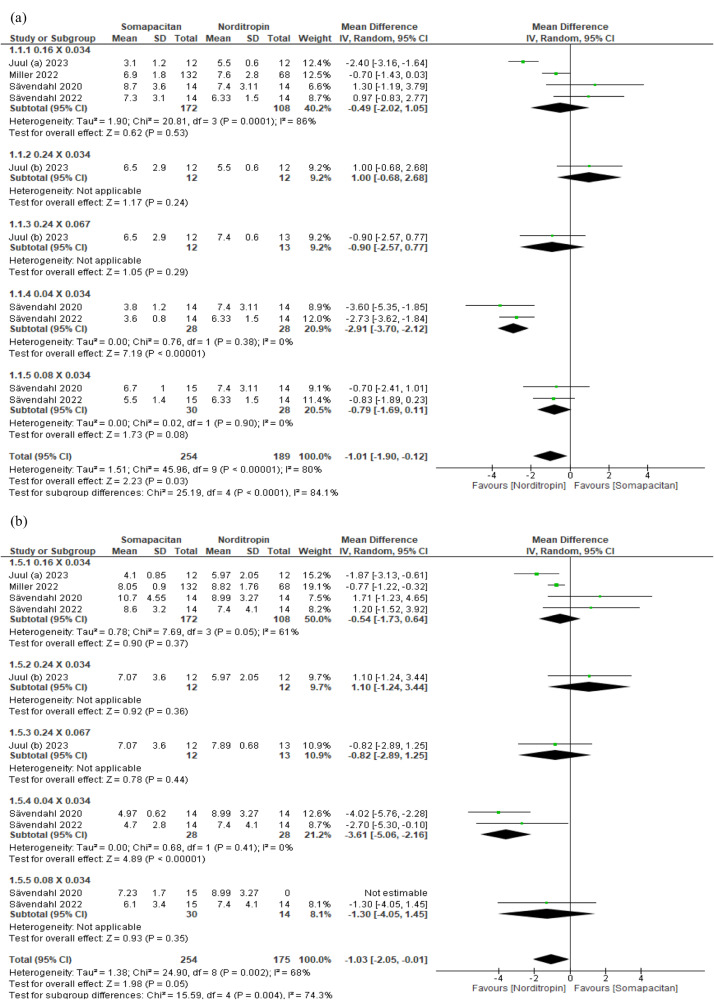
**a** Comparison between different doses of somapacitan and Norditropin in the effect on height velocity in children; **b** Comparison between different doses of somapacitan and Norditropin in the effect on height velocity-SDS in children

With regards to height velocity and height velocity SDS in children, no statistically significant difference was observed between different doses of somapacitan (0.04, 0.08, 0.16, and 0.24 mg/kg/week) and Norditropin (0.034, and 0.067 mg/kg/day) except for the 0.04 mg/kg/week somapacitan vs. 0.034 mg/kg/day Norditropin. In these doses, height velocity and height velocity-SDS favored Norditropin with an MD of −2.01 (95% CI: −3.7 to −2.12, *p* < 0.00001) and −3.61 (95% CI: −5.06 to −2.16, *p* < 0.00001), respectively. No heterogeneity among the studies was observed (Fig. 3a, b).

Moreover, we did not observe any significant differences between Norditropin (0.034 mg/kg/d) and somapacitan (0.16 mg/kg/wk) for the injection site reactions and adverse events OR of 0.9 (95% CI: 0.43–1.86, *p* = 0.77) and OR of 0.89 (95% CI: 0.51–1.57, *p* = 0.0.69), respectively, without heterogeneity (Supplementary Figs. 1 and 2).

### Adult population

**Fig. 4:**
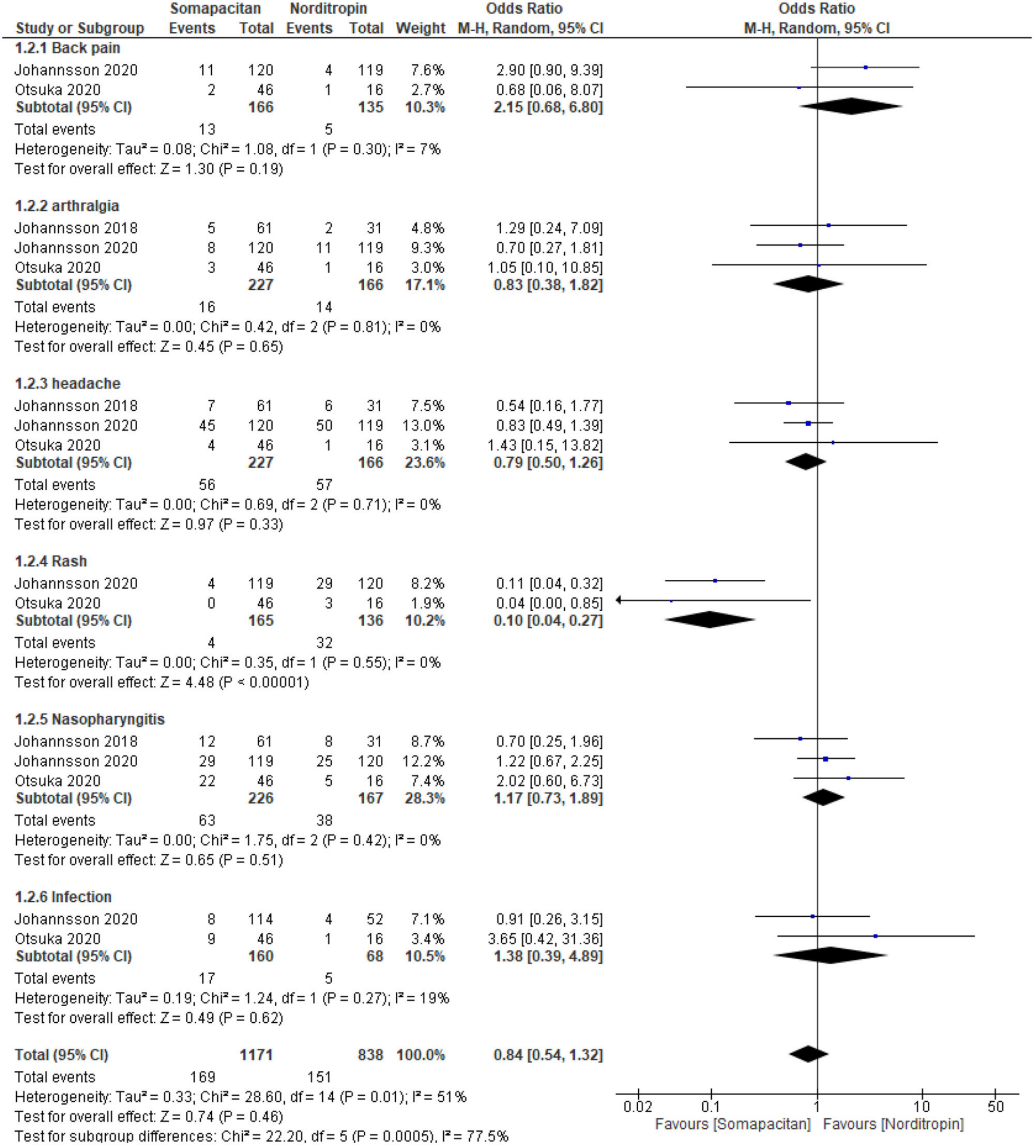
Comparison between somapacitan and Norditropin use in adults regarding adverse events

Regarding adverse events, no statistically significant difference was observed between Norditropin and somapacitan in adults (back pain, arthralgia, headache, nasopharyngitis, and infection), except for rash, which was statistically significantly associated with Norditropin use as somapacitan had less odds to cause rash with OR 0.1 (0.04–0.27, *p* < 0.00001) with no heterogeneity (Fig. 4).

**Fig. 5:**
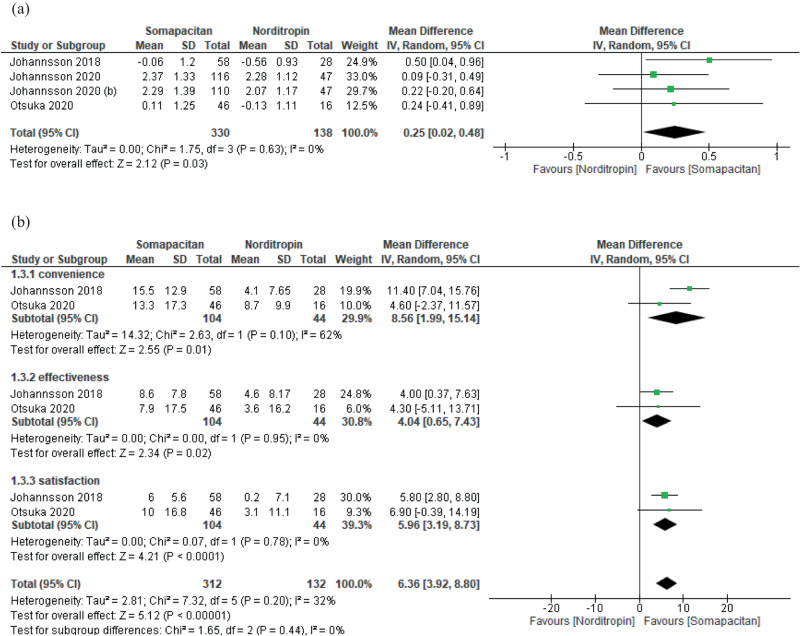
**a** Comparison between the adult patients taking Norditropin and somapacitan in IGF-1 SDS; **b** Comparison between the adult patients taking Norditropin and those taking somapacitan in TSQM-9 scale

Moreover, the mean change in IGF-1 SDS was statistically significantly higher in the somapacitan group compared to the Norditropin group with an MD of 0.25 (95% CI: 0.02–0.48, *p* = 0.03) and no heterogeneity (Fig. 5a). The overall TSQM-9 questionnaire score with its three domains (convenience, effectiveness, and satisfaction) was statistically significantly higher in the somapacitan group compared with the Norditropin group with an overall OR of 6.36 (95% CI: 3.92–8.8, *p* < 0.00001), with non-significant heterogeneity (Fig. 5b).

## Discussion

This systematic review aimed to (investigate the efficacy and safety of somapacitan versus daily Norditropin in children and adolescents with GH deficiency). Overall, no statistically significant distinction emerged between separate doses of Norditropin and somapacitan in terms of height growth velocity, with the exception of the 0.04 mg/kg/week somapacitan dose versus the 0.034 mg/kg/day Norditropin dose. Furthermore, patients receiving somapacitan displayed elevated IGF-1 levels compared to those receiving Norditropin. Somapacitan was found to be more convenient, effective, and satisfactory compared to Norditropin, as indicated by the TSQM-9 scores. Additionally, both GH treatments exhibited favorable safety profiles, although a higher prevalence of rash was observed in the Norditropin group, attributed to delayed hypersensitivity reactions. These findings contribute to a deeper understanding of the comparative efficacy, safety, and physiological impact of somapacitan and daily Norditropin treatments for GH deficiency in children and adolescents.

GH analogs have been used for persons with GH deficiency for over twenty years in adults [24] and more than fifty years in children [25]. Norditropin is a daily GH produced by recombinant DNA technology unlinked to a carrier protein. This would distinguish it from somapacitan, which is linked to albumin [13]. Although Norditropin has acquired popularity and significant benefits for treating GH insufficiency, it has drawbacks. For example, contemplating the administration of Norditropin analog for about 5–10 years, its once-daily consumption may cause significant discomfort to the user (particularly for children and adolescents) [26, 27]. Furthermore, its usage is not without adverse reactions, including rash and pain at injection spot, temporary fever, joint issues (stiffness, arthralgias), myalgias, paresthesias, peripheral edema (with fluid retention), gynecomastia, carpal tunnel syndrome, sleep apnea, sleep disorders, dyspnea, intracranial hypertension, insulin resistance (diabetes mellitus), scoliosis worsening, slipped capital femoral epiphysis [28–30]. Notably, though it has been postulated that GH supplementation can be associated with an increased risk of neoplasms based on in vitro observations, the association has not been shown. However, it has been recognized that if there is an already existing tumor, GH supplementation can unmask it if administered in the setting of GH deficiency [28]. Though it is from 2011, it does not suggest an increased risk of neoplasms [29]. Thus, we evaluated the influence of once-daily Norditropin and once-weekly somapacitan on height growth velocity, side effects, and overall satisfaction.

Regarding height growth velocity in children, no statistically significant distinction emerged between separate doses of Norditropin and somapacitan, except for 0.04 mg/kg/week of somapacitan versus 0.034 mg/kg/day of Norditropin. However, a pooled analysis revealed that the Norditropin group grew faster than the somapacitan. Although this finding contradicts previous studies that found comparable efficacy in somapacitan and Norditropin [31, 32], it can be explained through the hypothesis that the application of a long-acting GH is less physiological than daily GH injections (25–50 μg/kg/day) [33] and that constantly high GH levels may result in downregulation and perhaps less growth, influencing final height [34]. Still, the overall number of patients included in the analysis is relatively small to generalize such a conclusion.

Patients taking somapacitan have greater IGF-1 levels than those taking Norditropin. This is critical because IGF-1 is the principal mediator of GH and plays a critical role in controlling growth and metabolism in the human body [35]. Our analysis also highlighted the importance of IGF-1 synthesis in stimulating skeletal growth, with the change in IGF-1 levels accounting for a substantial portion of the variance in height growth. According to the literature, excess or insufficient IGF-1 and GH can cause growth issues such as gigantism, growth retardation, and short height. Children with short height but not GH deficiency were medicated with GH or not treated in some randomized, controlled, multicenter clinical trials [36, 37]. Over 5 years, the GH recipients’ height increased significantly in a dose-response pattern. Compared to untreated children, the change in IGF-1 levels from foundation accounted for the most variance (28% of the total variance) in greater height, suggesting the critical role of IGF-1 synthesis in stimulating skeletal growth. In both sexes, the IGF-1 impact was highly related to height growth [38].

Furthermore, the link between IGF-1 and GH is critical for maintaining skeletal muscle and lean body mass, decreasing adiposity, increasing immunological function, improving learning and memory, and increasing cardiovascular function, which cannot be expected in GH and IGF-1 deficit [39]. A study, however, found that IGF-1 levels showed low diagnostic accuracy as a screening test for GHD (due to the multifaceted nature of the disease). As a result, IGF-1 should not be used alone to screen for GHD [40].

According to the TSQM-9, somapacitan was statistically significantly more convenient, effective, and satisfactory than the Norditropin group. It is connected with fewer injections encountered by individuals in the somapacitan group than the Norditropin group. A comprehensive analysis of thirteen trials found that patients transitioning to less frequent injections enjoyed greater convenience and fulfillment, greater compliance rates, fewer adverse reactions, and improved quality of life across various diseases, including GH deficiency. Injections given less frequently are at least as effective as daily therapy [41].

Both Norditropin and somapacitan have a reasonably favorable safety profile regarding side events. However, the rash is more prevalent in the Norditropin group. The patient developed delayed hypersensitivity rashes to Norditropin, which is cell-mediated rather than IgE-mediated [42]. The rash was also found to reduce adherence in the Norditropin group [43].

This systematic review and meta-analysis possess several strengths. We evaluated different doses and side effects associated with administering Norditropin or somapacitan. Also, our study’s comprehensive evaluation of parameters beyond traditional clinical outcomes, encompassing adherence and quality of life considerations, reflects a holistic approach to assessing treatment effectiveness. However, our study is accompanied by noteworthy limitations. Variability in trial protocols, blinding methodologies, and randomization practices across included studies might introduce biases. Heterogeneity among selected trials, stemming from patient characteristics and demographic variations, necessitates careful interpretation due to potential influences on treatment responses. Constraints related to exploring dose-response relationships stem from data scarcity within specific dosage ranges. Lastly, the generalizability of our findings to diverse clinical settings merits consideration, recognizing that despite the study’s significance, applicability to different patient populations, treatment regimens, and dosages remains subject to inherent variations.

## Conclusion

Norditropin and Sogroya demonstrated similar effectiveness and safety characteristics. Patients’ quality of life can contribute to swapping the medications based on physician recommendations. Hence, future research efforts would significantly benefit from real-world mid- and long-term studies involving larger population samples. Furthermore, conducting cost-effectiveness studies is essential to assess the economic implications and potential advantages of using the therapies from the healthcare payers’ perspective.

### Supplementary information

Supplementary Information
