# Supplementary material for: Efficacy, safety, and patient satisfaction of norditropin and sogroya in patients with growth hormone deficiency: a systematic review and meta-analysis of randomized controlled trials
Source: Endocrine. 2024 Apr 24;85(2):545–57. doi: 10.1007/s12020-024-03834-z (PMC11291597; doi:10.1007/s12020-024-03834-z)

**Supplementary files for:**

**Efficacy, Safety, and Patient Satisfaction of Norditropin and Sogroya in Patients with Growth Hormone Deficiency: A Systematic Review and Meta-Analysis of Randomized Controlled Trials**

**Authors:** Obieda Altobaishat^1^, Mohamed Abouzid^2,3*^, Mostafa Hossam El Din Moawad^4,5^, Abdulrahman Sharaf^6^, Yazan Al-Ajlouni^7^, Tungki Pratama Umar^8,9^, Abdallah Bani-salameh^1^, Mohammad Tanashat^10^, Omar Abdullah Bataineh^1^, Abdulqadir J. Nashwan^11^.

**Affiliations:**

1. Faculty of Medicine, Jordan University of Science and Technology, Irbid, Jordan.
2. Department of Physical Pharmacy and Pharmacokinetics, Faculty of Pharmacy, Poznan University of Medical Sciences, Rokietnicka 3 St., 60-806, Poznan, Poland.
3. Doctoral School, Poznan University of Medical Sciences, 60-812, Poznan, Poland.
4. Faculty of Pharmacy, Clinical Department Alexandria University, Alexandria, Egypt.
5. Faculty of Medicine, Suez Canal University, Isamailia, Egypt.
6. Department of Clinical Pharmacy, Salmaniya Medical Complex, Government Hospital, Manama, Bahrain.
7. School of Medicine, New York Medical College, New York, USA.
8. Faculty of Medicine, Universitas Sriwijaya, Palembang, Indonesia.
9. Division of Surgery and Interventional Science, University College London, London, United Kingdom
10. Faculty of Medicine, Yarmouk University, Irbid, Jordan.
11. Hamad Medical Corporation, Doha, Qatar.

Supplementary Figure 1. Comparison between somapacitan and Norditropin for the injection site reactions.


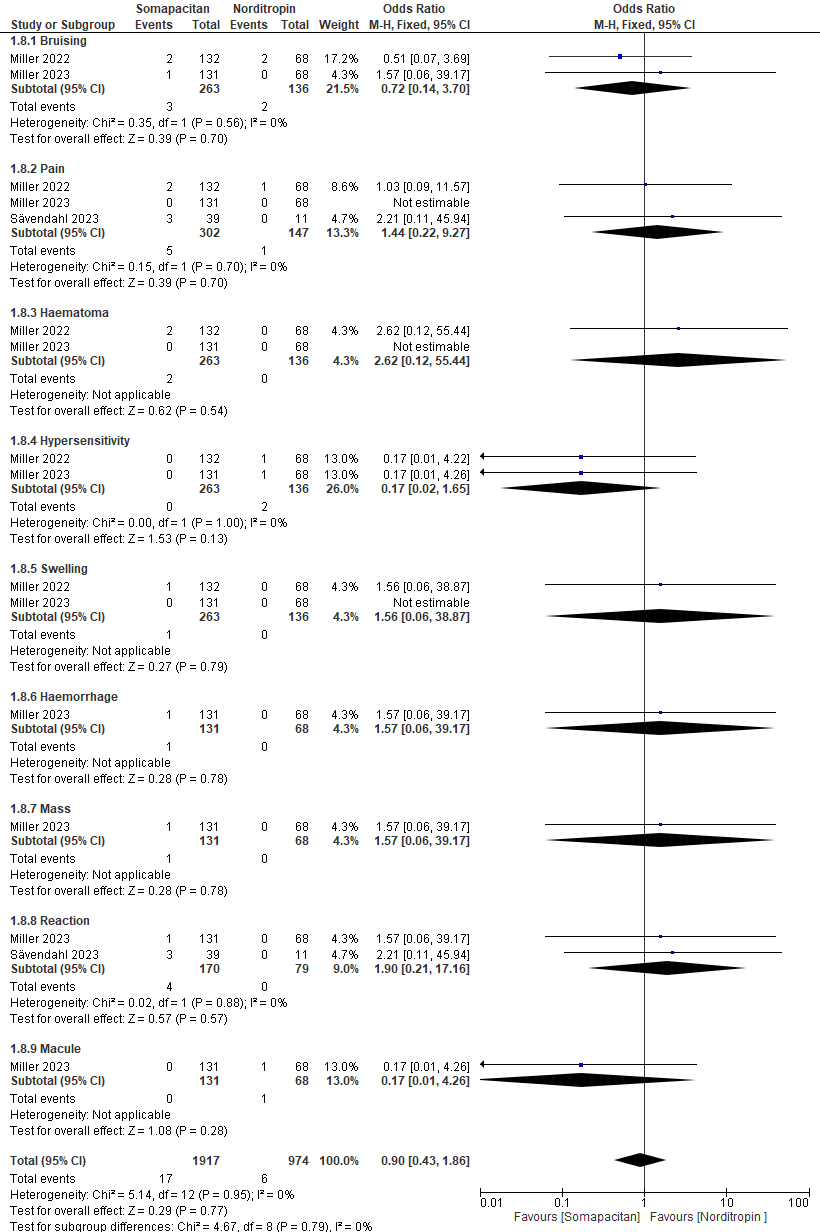


Supplementary Figure 2. Comparison between somapacitan and Norditropin for the various side effects in children.


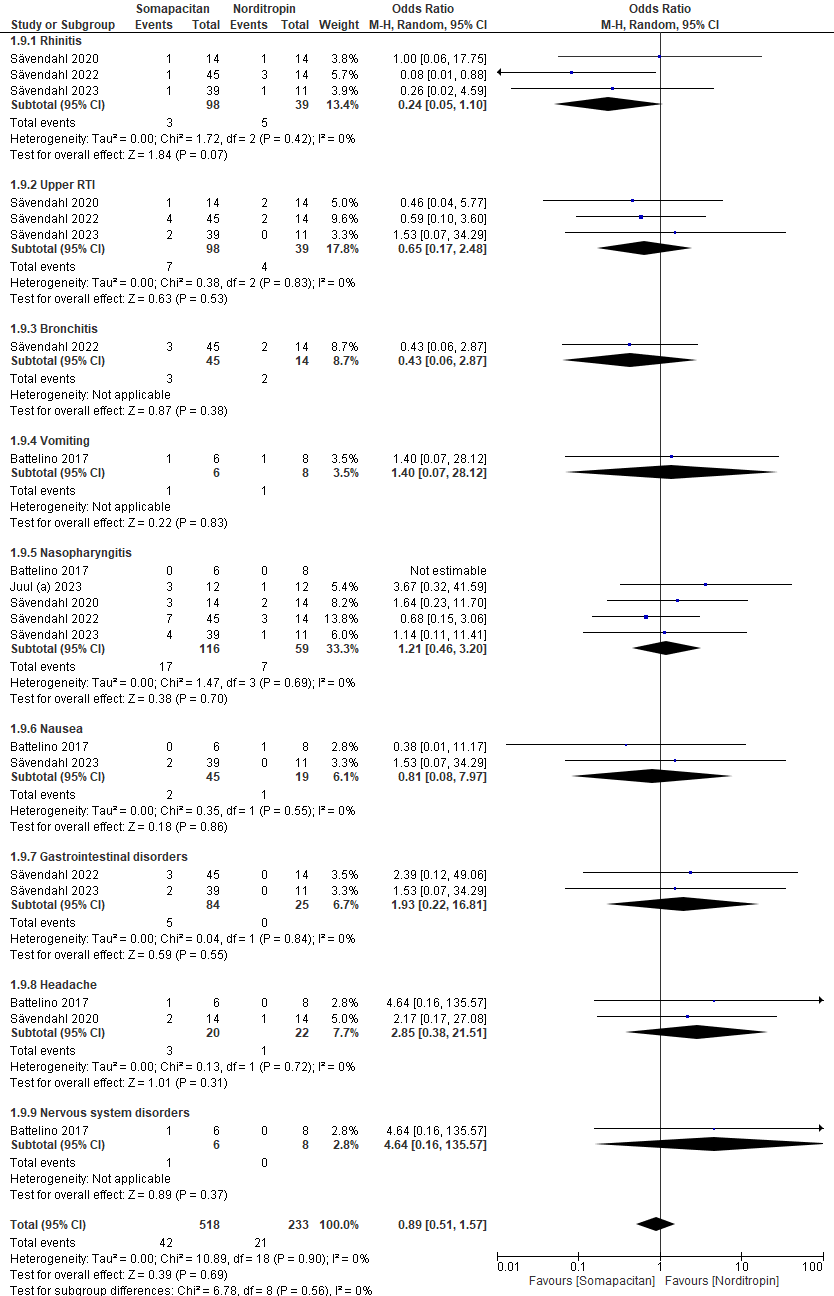

Supplement: Supplementary file 1 — Supplementary Information [file 12020_2024_3834_MOESM1_ESM.docx]
